# Assessing quality of life among elementary school students: Validation of the Korean version of the Meaning in Life in Children Questionnaire

**DOI:** 10.3389/fpsyg.2022.904115

**Published:** 2022-08-04

**Authors:** Younyoung Choi, Joo Yeon Shin

**Affiliations:** ^1^Department of Psychology, Ajou University, Suwon, South Korea; ^2^Graduate School of Education, Inha University, Incheon, South Korea

**Keywords:** meaning in life, children, reliability, validity, psychometric property

## Abstract

Meaning in life (MIL) has been widely recognized as a hallmark of psychological well-being and positive youth development. The goal of this study was to validate the Korean version of the Meaning in Life in Children Questionnaire (K-MIL-CQ) utilizing the framework suggested by the Standards for Educational and Psychological Testing. Data were obtained from 277 fifth graders aged 10–11 in three elementary schools in Seoul and Gyeonggi through a paper-and-pencil survey (55.2% boys). We translated the MIL-CQ, a 21-item self-report measure developed based on Frankl’s “meaning triangle,” into Korean. Psychological well-being measures were also assessed. Validity and reliability data were collected. (1) The content of domains and items was appropriate for measuring MIL among children. (2) A three-factor model consisting of attitude, creativity, and experience pathways was extracted *via* exploratory factor analysis, and a three-factor hierarchical model including attitude, creativity, and experience as first-order factors and MIL as a second-order factor was confirmed *via* confirmatory factor analysis. (3) Higher levels of MIL were related to higher levels of satisfaction with life, self-esteem, positive affectivity, and lower levels of negative affectivity. (4) All item fit statistics were acceptable based on the Rasch model. (5) The analysis of the measurement invariance of each item showed that the responses to one item varied by gender, suggesting that additional items might facilitate better measurement of MIL in children. This study provides validity and reliability evidence that K-MIL-CQ is appropriate for measuring MIL among South Korean elementary school students.

## Introduction

Meaning in life (MIL) has been widely recognized as a hallmark of quality of life and psychological, physical, and spiritual well-being (for reviews, see Steger et al., 2006; Steger, 2009, 2012; Hicks et al., 2010; Park, 2012; Steger and Shin, 2012; Heintzelman and King, 2014) as well as one of the critical components of positive youth development (Damon et al., 2003) and positive education (Seligman et al., 2009). Research on MIL has surged over the past two decades, generating diverse conceptual models and definitions along with various assessment tools. For example, Frankl’s theory of logotherapy (Frankl, 1959) suggests three pathways for discovering or creating MIL using the framework of the “meaning triangle”: the creative, experiential, and attitudinal pathways. Instruments such as the Purpose in Life Test (Crumbaugh and Maholick, 1964) and the Life Purpose Questionnaire (Hutzell, 1989) have been utilized to assess logotherapy-related concepts. Meanwhile, Steger et al. (2006) proposed that MIL consists of comprehension and purpose, which are reflected in the Meaning in Life Questionnaire. In addition, George and Park (2016) developed the Multidimensional Existential Meaning Scale, which includes one’s life with value and significance (mattering), having a broader purpose in life, and one’s life being coherent and making sense (comprehension; Leontiev, 2005; Reker and Wong, 2012; Heintzelman and King, 2014; Martela and Steger, 2016).

Despite the recent abundance of conceptual and empirical discourse, previous studies on MIL have mostly focused on older adolescents, adults, or aging populations. The topic of MIL during early adolescence (generally defined as the period from 10 to 14 years; elementary school students) has not been fully investigated. One reason hindering the empirical understanding of young adolescents’ MIL might be the lack of reliable and valid instruments. Shoshani and Russo-Netzer (2017) posited that existing measures contain highly abstract and general formulations of MIL; these are not appropriate for use with young adolescents because the items are too difficult for them to understand. Accordingly, the MIL in Children Questionnaire (MIL-CQ) was developed to facilitate accurate assessment of MIL in children aged 9–12 (Shoshani and Russo-Netzer, 2017). In this study, we sought to validate the Korean version of the MIL-CQ to facilitate robust empirical research on MIL in children.

The original MIL-CQ has its foundation in Frankl’s (1959) “meaning triangle” pathways, namely, creative, experiential, and attitudinal. These pathways have been utilized in intervention protocols with both children and adults dealing with mental health difficulties or challenging life events such as severe physical illness (Greenstein and Breitbart, 2000; Kang et al., 2013), as well as a wide range of cognitive, interpersonal, behavioral, and emotional experiences related to meaning in daily life in a relatively concrete manner (Shoshani and Russo-Netzer, 2017). According to Shoshani and Russo-Netzer (2017), the first subscale of the MIL-CQ captures the creative pathway toward discovering MIL, which assesses children’s sense of meaning developing from their self-concordant actions, deeds, everyday behaviors, and habits. The experience subscale concerns children’s sense of connection to family, friends, all humans, or to transcendent states and entities such as nature, art, beauty, God, or a supreme power. Finally, the attitude subscale addresses whether children approach difficult or painful life experiences from a positive perspective.

Researchers, educators, and mental health practitioners have suggested that the topic of MIL among young adolescents should be given more attention for the following reasons. First, the literature supports the notion that MIL facilitates identity formation, which is a major developmental task in early adolescence. For instance, a recent longitudinal study found a bidirectional, mutually reinforcing relationship between MIL and identity development (Negru-Subtirica et al., 2016). Meaning in life has also been shown to serve as a mediator of ethnic identity and adjustment among adolescents from Latin, Asian, and European American backgrounds (Kiang and Fuligni, 2010). Second, the role of MIL in positive youth development is explicitly emphasized in the PERMA-H model of human happiness and well-being (i.e., positive emotion, engagement, relationships, meaning and purpose, achievement, and health), proposed in positive education, which is the application of positive psychology in educational settings (Seligman, 2011; Norrish et al., 2013; Lai et al., 2018).

Third, a substantial amount of research has demonstrated that MIL serves as an important index of mental health and is a crucial protective factor in various psychological problems among youth, such as depression, delinquency, and suicide. Studies have shown that mental health issues such as suicide, depression, and a low sense of happiness and well-being are becoming increasingly severe among South Korean adolescents, with an earlier age of onset. For instance, the prevalence of suicidal thoughts is high, and the rate of suicide attempts is increasing among younger age groups. A 2018 survey that sought to compute a happiness index for South Korean children and adolescents reported that the prevalence of suicidal impulsivity in elementary school students was 20.7% (increased from 14.3% in 2015), and that the number of high-risk students, who experienced suicidal impulsivity three or more times a week, has been increasing each year (Youm and Kim, 2018). A more recent South Korean study of 573 elementary school students (5th–6th grade) reported that 5.1% had established a suicide plan, 3.0% had made a suicide attempt, and 7.1% exhibited suicidal behavior (Kim, 2019), emphasizing the severity of the issue of suicide in this population. Depression has also been reported among this population, and a study analyzing data from 2,210 6th graders in elementary schools (the 3rd Korean Children and Youth Panel Survey, 2010) reported that depression mostly affected life satisfaction (Kim, 2014). However, this study only included self-esteem as an individual protective factor.

Although MIL has been identified as an important individual protective factor linked with these mental health issues, existing studies have mainly focused on factors such as parental support, teacher support, peer support, self-esteem (e.g., Lee and Heo, 2003; Kim, 2014), optimism, and emotion regulation (e.g., Kwon, 2019) in understanding suicide or depression among children, leaving out the role of MIL in this population. The experiences of elementary school students in higher grades, who have just entered puberty (grades 4–6), entail more rapid physical and psychological changes than other life stages as well as the formation of self-identity through confusion, conflict, and stress (Kwon and Yang, 2014). To facilitate positive mental health and development in young adolescents, more empirical research on MIL is warranted.

The limited research on MIL among young adolescents is possibly owing to the belief that individuals in this developmental period lack the psychological maturity to understand the abstract and complex aspects of MIL (Brassai et al., 2011; Shoshani and Russo-Netzer, 2017). However, an increasing number of studies have posited that MIL can and should be examined in young adolescents because children can articulate their experience of MIL, for example, describing sources of personal meaning, similar to college students (De Volger and Ebersole, 1983; Taylor and Ebersole, 1993). Steger et al. (2013) also proposed a model of lifelong meaning, emphasizing the developmental aspects of MIL formation in early adolescence. Recent South Korean studies also support this notion. Through a logotherapy-based content analysis of the experiences of MIL among 1,600 higher-grade elementary school students (aged 11–13), Kang (2017) concluded that this population can understand the concept of MIL. The themes of the students’ MIL experiences identified in this study were “important things in my life,” “activities I like to do,” “experiences that make me feel like I am loved,” “times when I feel grateful,” and “importance of choices.” Lim (2020) also examined the perceptions of MIL among young adolescents and their parents, reporting that these students were able to identify and appraise their sense of meaning. Furthermore, several studies have successfully adopted the concept of MIL in examining the effects of life-respect education programs on MIL among elementary school students (e.g., Ryu and Lee, 2008). These studies consistently support the notion that the MIL construct is applicable to young adolescents.

Another reason hindering further empirical understanding of young adolescents’ MIL might be the lack of reliable and valid instruments. To the authors’ knowledge, the only scale available for measuring MIL in children in South Korea is the Meaning of Life Scale (Kang et al., 2007). Like the original MIL-CQ, this scale, developed for higher-grade elementary school students (grades 4–6), is based on Frankl’s (1959) conceptual framework of logotherapy. However, the Meaning of Life Scale comprises 24 items with five sub-factors (i.e., relational experience, positive attitude, satisfaction/hope, pursuit of goals, and experience of family love). This hinders an accurate understanding of MIL because there is a risk of over-inclusion of other constructs, such as satisfaction, hope, and love, which usually overlap with the construct of MIL but are still viewed as distinct from it. Further, this scale includes one item directly related to death, blurring the conceptual boundary of the MIL construct. The applicability of this scale is also limited because it is only used in the South Korean context, mostly in nursing, and does not exactly match Frankl’s three components of meaning, making it difficult to use it to compare the levels of MIL in children from various countries. Thus, there is a need for a scale that reflects Frankl’s theoretical framework, validated in the South Korean context, which will also enable cross-cultural comparisons of MIL.

Culture influences individuals’ conceptions and experiences of well-being and happiness, including MIL. Individuals make judgments regarding their lives, such as what is important in their lives, what constitutes a meaningful life, and how to achieve meaning in life, based on their cultural values. Literature on cultural differences has posited that East Asian culture commonly emphasizes vertical collectivistic values, where observing hierarchies, showing respect for the elderly, and meeting parents’ expectations have strong significance in one’s life (Triandis and Gelfand, 1998; Steger et al., 2008). In addition, South Korean society believes fervently in a university-based meritocracy (Cho, 2016), emphasizing the strong ties between educational achievement, specifically a prestigious college education, and one’s social rank, upward mobility, and career success (Garrison et al., 2017). Considering that a society’s specific cultural aspects could influence MIL experiences, it is important to investigate the psychometric properties of the MIL-CQ with a sample of Korean children, given that the original scale was developed in Israel.

### The present study

The broad aim of this study was to facilitate research on MIL in early adolescence. To that end, we undertook validation of the Korean version of the MIL-CQ, originally developed by Shoshani and Russo-Netzer (2017), based on the framework suggested by the Standards for Educational and Psychological Testing (AERA et al., 2014). Specifically, we first collected evidence related to content validity through a domain analysis by subject matter experts and by computing the content validity index (CVI). Second, we investigated the internal structure of the MIL-CQ (i.e., construct validity) by conducting exploratory analysis. Then, we examined the model fit statistics of the hierarchical factor model with the three factors (attitude, creativity, and experience) as first-order factors and MIL as a second-order factor by confirmatory factor analysis. Third, we determined criterion validity by examining correlations with the Satisfaction with Life Scale (SWLS), Positive and Negative Affect Schedule (PANAS), and the Rosenberg Self-Esteem Scale (SES). Relying on previous studies that consistently support the positive relationships between MIL and subjective well-being indictors (King et al., 2006) and a favorable sense of self (Steger et al., 2006), it was hypothesized that MIL is positively correlated with life satisfaction, positive affect, and self-esteem and negatively correlated with negative affect. Fourth, we evaluated the item fit of each item using the Rasch model (Linacre, 2004). Fifth, we examined the measurement invariance of each item by gender using differential item functioning (DIF, Osterlind and Everson, 2009) analysis based on the item response theory (IRT; Thissen et al., 1993; Embertson and Reise, 2000). Lastly, reliability was evaluated using Cronbach’s alpha and standard error of measurement (SEM) based on the IRT (Lord, 1980).

## Materials and methods

### Participants

A total of 277 students participated in the paper-and-pencil survey. They were recruited from three elementary schools located in Seoul and Gyeonggi province during the fall semester of 2017. All children were 5th graders aged 10–11 years (55.2% boys and 44.8% girls). Students and their parents were informed of the voluntary and anonymous nature of participation, with no compensation/advantage or disadvantage to their grades. Class teachers administered the questionnaire, which took ~15 min to complete. With parental consent, students who provided informed consent were administered the survey.

### Procedure

First, after obtaining permission from the authors of the original MIL-CQ *via* personal email communication (Shoshani and Russo-Netzer, 2017), the tool was translated into Korean by the corresponding author and one counseling psychologist, both of whom held PhDs from universities in the United States. Then. The Korean version was blindly back-translated by a bilingual expert and compared with the original English tool to examine whether the translated version accurately reflected the original items. The final Korean version was evaluated by one elementary school teacher and one school counselor who had worked with elementary school students in South Korea to ensure comprehensibility.

The researchers contacted three elementary schools located in Seoul and Gyeonggi province and asked 5th grader teachers to administer the survey in their class. Teachers who agreed to participate in the current study were individually contacted by the researchers and provided detailed guidance regarding the survey procedure. The teachers in charge of administering the questionnaires attended a telephone orientation session with the researchers. The teachers were specifically guided to allocate sufficient time to administer the questionnaires, such as using a homeroom class. The teachers were also asked to maintain quiet and organized classroom conditions to help students stay focused on completing the questionnaire. Students were guided to choose the answer that best described them and specifically informed that there were no right or wrong answers. To minimize the risk of misunderstandings of the meanings of survey questions, teachers were also trained to provide brief answers when asked to clarify survey items. A total of 277 students were recruited by teachers and all the 277 responses from the students were used for analyses. There were no missing data.

### Instruments

#### Korean version of the MIL-CQ

Participants’ sense of MIL was measured using the Korean version of the MIL-CQ (K-MIL-CQ; Shoshani and Russo-Netzer, 2017). The MIL-CQ consists of 21 items measuring three subscales of meaning, namely, creative (eight items), experience (seven items), and attitude (six items). Participants responded to each item on a five-point Likert scale ranging from 1 (not at all) to 5 (to a very large extent). The scale has been reported to have sound psychometric properties with evidence of validity and reliability (Shoshani and Russo-Netzer, 2017). The internal consistency, as demonstrated by Cronbach’s alpha, was 0.82 in the original study (Shoshani and Russo-Netzer, 2017) and 0.94 in the current study.

#### Korean version of SWLS

Participants’ global sense of life satisfaction was measured using the five-item Korean version of the SWLS (K-SWLS; Cho and Cha, 1998; Lim et al., 2010). The original five-item SWLS, developed by Diener et al. (1985), has good psychometric properties. Participants were asked to respond to each item on a five-point Likert scale ranging from strongly disagree (1) to strongly agree (5). Higher scores indicate higher satisfaction with life. A validation study was conducted using a sample of Korean adolescents (Lim, 2012). The Cronbach’s alpha was 0.86. The scale’s concurrent validity was established by showing the association of the K-SWLS with the measures of emotional well-being (*r* = 0.51), social well-being (*r* = 0.51), and psychological well-being (*r* = 0.54). In the current study, Cronbach’s alpha was 0.86.

#### Korean version of PANAS

The Korean version of the PANAS (K-PANAS; Lee et al., 2003) was used to measure participants’ emotional well-being. The PANAS, originally developed by Watson et al. (1988), consists of 20 items grouped into two subscales: positive affect (PA; 10 items) and negative affect (NA; 10 items), and rated on a five-point Likert scale ranging from 1 (very slightly or not at all) to 5 (extremely). Higher PA scores indicated greater intensity of positive emotions, and higher NA scores indicated greater intensity of negative emotions. In several studies (e.g., Lee and Lee, 2007; Kim and Kim, 2012), short sentences incorporating each adjective to facilitate children’s understanding of their usage were included. This technique of incorporating explanatory sentences was utilized. The K-PANAS has shown good psychometric properties in previous studies (Lee et al., 2003; Lee and Lee, 2007; Kim and Kim, 2012). Cronbach’s alpha in previous studies were 0.84 for PA and 0.87 for NA. In the current study, the alpha coefficients were 0.78 for PA and 0.86 for NA.

#### Korean version of SES

Self-esteem, defined as a positive evaluation or judgment of the self (Rosenberg, 1965), was assessed using the Korean version of the Rosenberg SES (K-SES; Lee and Won, 1995). This 10-item tool is rated on a five-point Likert scale ranging from 1 (strongly disagree) to 5 (strongly agree). Higher scores were indicative of higher self-esteem. Suitable psychometric properties have also been reported (Tippett and Silber, 1965). Internal consistency was 0.85–0.88 in the original study (Rosenberg, 1965), 0.83–0.87 in Park and Sohn’s (2019) study, and 0.85 in the present study.

### Data analysis

Descriptive statistics of all measures were investigated using the mean, standard deviation, skewness, and kurtosis. Reliability and validity were computed to evaluate the psychometric properties of the Meaning of Life Scale. Reliability was evaluated using Cronbach’s alpha as internal consistency and standard measurement errors based on IRT (Lord, 1980). Validity evidence was collected based on the Standards for Educational and Psychological Testing (AERA et al., 2014), which recommends that a validation study should collect evidence on content, internal structure, relations with other measures, response pattern to each item, and consequences.

In this study, evidence based on content (i.e., content validity) was collected through domain analysis by subject matter experts. A total of five experts participated in rating adequacy of each factor and items of the K-MIL-CQ, all of whom hold doctoral degrees in counseling psychology and have extensive experience in research and practice regarding youth mental health and positive development. Evidence based on internal structure (i.e., construct validity) was collected by EFA and CFA with the Geomin (oblique rotation) with the WLSMV estimator using Mplus (Muthén and Muthén, 1998–2007). Evidence based on relationships with other measures (criterion validity) was evaluated by computing the correlation values with the K-SWLS, K-PANAS, and K-SES. Evidence based on the response process of each item was collected by evaluating item fit using the Rasch model by WinSteps (Linacre, 2004) Finally, and DIF was computed for each item in terms of gender using IRTpro (Cai et al., 2011). Differential item functioning were detected using Wald tests with accurate item parameter error variance–covariance matrices computed using the Supplemented EM algorithm (see IRTpro manual).

## Results

### Descriptive statistics and correlation analysis

Table 1 shows the mean, standard deviation, skewness, kurtosis, and correlation coefficient values between the measures. The skewness and kurtosis values of all measures satisfied the normal distribution assumption (Byrne, 2001). The K-MIL-CQ showed a strong positive correlation with the K-SWLS, K-SES, and PA subscale of the K-PANAS (*r* = 0.67, *r* = 0.70, *r* = 0.65, respectively), while it showed a moderate negative correlation with the NA subscale of the K-PANAS (*r* = −0.32).

**Table 1:** Correlation values between measures, means, standard deviations, skewness, and kurtosis.

	K-MIL-CQ	K-SWLS-C	K-PANAS (Positive)	K-PANAS (Negative)	K-SES
K-MIL-CQ	–				
K-SWLS-C	0.67^**^	–			
K-PANAS (PA)	0.65^**^	0.54^**^	–	–	
K-PANAS (NA)	−0.32^**^	−0.44^**^	−0.13^*^	–	
K-SES	0.70^**^	0.69^**^	0.44^**^	−0.53^**^	–
Mean	82.34	20.23	35.41	25.09	37.46
SD	14.79	4.70	6.58	8.71	8.12
Skewness	−0.61	−0.92	−0.33	0.12	−0.51
Kurtosis	−0.11	0.21	0.20	−0.78	−0.28

* *p* < 0.05 and

** *p* < 0.01.

### Psychometric properties

#### Sources of validity evidence

##### Evidence based on contents

The CVI (Content Validity Index: Lynn, 1986; Polit et al., 2007) was computed to evaluate the five experts’ rating agreement of the five experts. The average CVI value across all items was 0.88; thus, most content experts agreed that the items were generally acceptable. Three items (items 7, 17, and 20) were modified because 2–3 experts considered it difficult for children to understand. Table 2 summarizes the expert opinions on each factor and items of the K-MIL-CQ.

**Table 2:** Content analysis of the K-MIL-CQ by five experts.

Opinions on each factor	
	All five experts assessed that the three theoretically suggested each factor adequately reflected the construct of meaning in life.
Opinions on items	
Item 7	Item contents: “I make up songs, stories, games, and other things that can contribute to others.” It was pointed out that the word “contribute” can be difficult to understand (by three experts).
Item 17	Item contents: “I enjoy the beauty in life.” It was pointed out that the expression “beauty in life” may be abstract and difficult for elementary school students to understand (by two experts).
Item 20	Item contents: “I feel connected to God or a higher power that gives me guidance.” It was pointed out that the concept of “God or a higher power” might be too abstract for elementary school students to understand (by three experts).

##### Evidence based on internal structure

**Figure 1:**
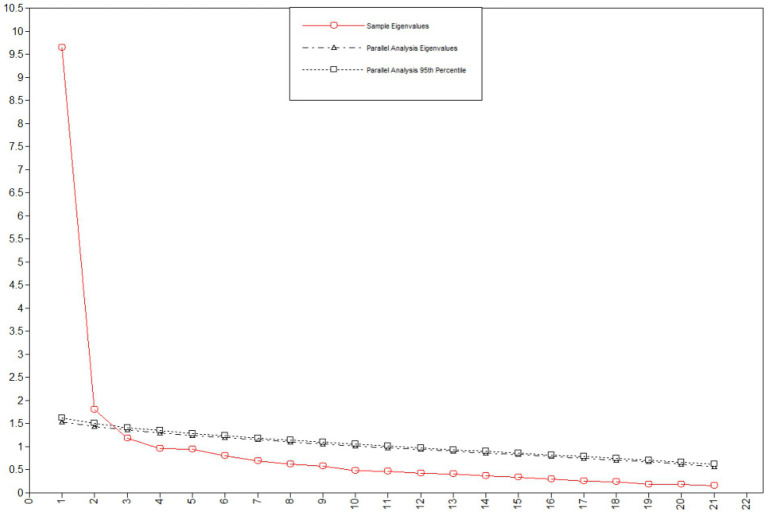
Scree plot and parallel analysis.

An EFA was conducted to evaluate the internal structure of K-MIL-CQ. First, we examined the Kaiser–Meyer–Olkin (KMO) measure of sampling adequacy (KMO = 0.94) and the results of Bartlett’s sphericity test (*p* < 0.0001), which indicated that the data were suitable for factor analysis. Next, we conducted EFA using the Geomin (oblique rotation) with the WLSMV estimator. Because all items are on a five-point Likert scale, we considered them all as categorical variables in the analysis. The scree plot suggested a three-factor solution, which accounted for 60.15% of the variance (Figure 1). The scree plot suggested three factors and the parallel analysis reports two factors. We decided the three-factor model as the final model based on the statistical results and the proposed model of the original questionnaire. The factor loadings of each item, determined through the EFA, are listed in Table 3. Most of the items were loaded into one of the three factors. However, Item 8 (“I like taking the time to do important and meaningful things”) did not load to a dominant factor. Items 20 (“I feel connected to God or a higher power that gives me guidance”) and 21 (“Being in nature makes me happy and calm”) loaded to the creativity factor in the current sample, though these items were expected to load to the experience factor in the original scale. These three items were excluded from the final scale because it is unclear whether they appropriately represent the creativity factor. The model fit statistics of the three-factor model showed an appropriate fit:comparative fit index (CFI) = 0.98, Tucker–Lewis index (TLI) = 0.96, root mean square error of approximation (RMSEA) = 0.07, and standardized root mean square residual (SRMR) = 0.04. The model fit statistics were evaluated based on established criteria that CFI and TLI should be >0.90; RMSEA should be <0.08; and SRMR should be <0.10 (Bentler and Bonett, 1980; Bentler, 1990; Browne and Cudek, 1993; Medsker et al., 1994).

**Table 3:** Items of K-MIL-CQ, factor loadings of EFA and standardized coefficients of second-order factor model.

Items		EFA			Second-order factor model	
		Attitude	Creativity	Experience	Standardized coefficient	SE
**Item 1**	**I think that even if something bad happens in my life, I can overcome it.**	0.815	0.098	−0.01	0.883	0.024
**Item 2**	**Even though bad things sometimes happen to me, I think life is beautiful.**	0.944	0.011	−0.026	0.922	0.016
**Item 3**	**Even though there are sad things in the world, I think life is worth living.**	0.853	0.070	0.024	0.931	0.016
**Item 4**	**think I can learn or gain something from experiences, even when I experience something bad.**	0.913	−0.027	−0.007	0.877	0.023
**Item 5**	**I think that even in negative experiences I can find something positive.**	0.812	−0.063	0.045	0.795	0.026
**Item 6**	**I accept the things I cannot change in myself or the world.**	0.311	0.001	−0.100	0.219	0.058
**Item 7**	**I make up songs, stories, games, and other things that can contribute(help) to others.**	−0.035	0.555	−0.025	0.459	0.049
Item 8	I like taking the time to do important and meaningful things.	0.238	0.388	0.224	–	–
**Item 9**	**I feel I do things that are beneficial to others.**	0.209	0.736	−0.047	0.866	0.019
**Item 10**	**I do things that are important for others.**	−0.02	0.902	0.024	0.870	0.019
**Item 11**	**I often do things to contribute and help others.**	0.008	0.777	−0.022	0.738	0.030
**Item 12**	**My actions help my environment and the world.**	0.004	0.824	0.036	0.829	0.026
**Item 13**	**I try to spend my free time doing meaningful things.**	0.291	0.533	0.015	0.795	0.029
**Item 14**	**I take actions that will help me achieve the goals in life that are important to me.**	0.237	0.562	0.091	0.841	0.022
**Item 15**	**Being with my family gives me strength.**	0.316	−0.057	0.586	0.805	0.03
**Item 16**	**My relationships with people my age makes me feel good.**	−0.030	0.086	0.885	0.870	0.025
**Item 17**	**I enjoy the beauty in life. (e.g., going to beautiful park, seeing trees and flowers, watching good movies, having a good time with family or friends)**	0.398	0.029	0.566	0.940	0.015
**Item 18**	**I feel happiness and joy when I am with people who are close to me.**	0.067	0.153	0.732	0.880	0.023
**Item 19**	**I like to travel and enjoy the beauty that exists in the world.**	−0.005	0.152	0.587	0.648	0.046
Item 20	I feel connected to God or a higher power that gives me guidance.	−0.007	0.5	0.096	–	–
Item 21	Being in nature makes me happy and calm.	0.013	0.389	0.299	–	–

The bold text denotes the items included in the final version of the Korean version of MIL-CQ. The Korean-translated version of this questionnaire is available upon request.

**Figure 2:**
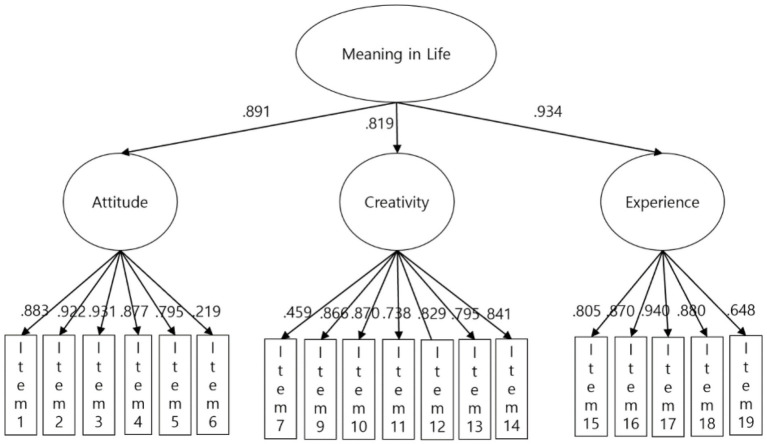
Second-order factor structure of K-MIL-Q.

Next, we conducted a hierarchical confirmatory factor analysis with the three factors (attitude, creativity, and experience) as first-order factors and MIL as a second-order factor (Figure 2). The standardized coefficient of all items in the model is shown in Table 3. The model fit statistics of the hierarchical second-order model showed an appropriate fit: comparative fit index (CFI) = 0.98, Tucker–Lewis index (TLI) = 0.98, root mean square error of approximation (RMSEA) = 0.07.

##### Evidence based on relations with other measures

We evaluated the criterion validity of the K-MIL-CQ by computing the correlation values with K-SWLS-C, K-SES, and K-PANAS (Table 1). The total K-MIL-CQ scores showed strong positive correlations with the total scores of the K-SWLS-C, K-SES, and PA subscale of the K-PANAS (*r* = 0.67, *r* = 0.70, *r* = 0.65, respectively), while showing a moderate negative correlation with the score of the NA subscale of the K-PANAS (*r* = −0.32).

##### Evidence based on response patterns

**Figure 3:**
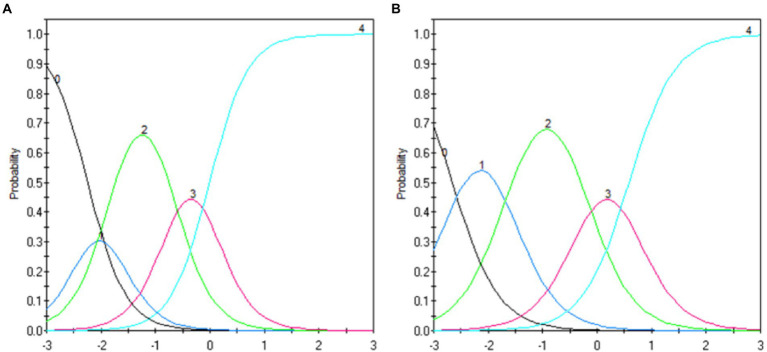
Exemplary probability response function curves (Items 1 and 9). Probability response function curve for **(A)** Item 1 and **(B)** Item 9.

The Rasch model (i.e., rating scale model) was applied to evaluate item fit by computing the infit and outfit statistics (Table 4). The infit and outfit scores of the final items were acceptable based on the criterion that a range of 0.6–1.4 depicts a good fit (Smith and Smith, 2004). In addition, a plot of the probability response function (PRF) for each item was created and evaluated (Figure 3 as two examples). The PRF suggested that all categories of each item contained the measurement information.

**Table 4:** Item difficulty parameters and standard errors, Infit and Outfit statistics in Rasch analysis, DIF analysis.

Item	Rasch model						DIF analysis	
	Difficulty		Infit		Outfit		*χ* ^2^	*p*
	*b*	SE	MNSQ	ZSTD	MNSQ	ZSTD		
Item 1	−0.26	0.08	0.79	−2.4	0.83	−1.5	0.80	0.97
Item 2	−0.26	0.08	0.81	−2.3	0.78	−1.9	11.00	0.05
Item 3	−0.51	0.08	0.72	−3.3	0.74	−2.1	2.70	0.74
Item 4	−0.28	0.08	0.85	−1.7	0.80	−1.7	4.50	0.48
Item 5	−0.11	0.08	0.97	−0.3	−0.88	−1.0	3.70	0.59
Item 6	0.23	0.07	0.95	0.12	1.40	0.17	1.93	0.23
Item 7	0.37	0.06	1.03	0.09	1.48	0.11	2.10	0.17
Item 9	0.29	0.07	0.64	−4.8	0.63	−4.3	4.90	0.43
Item 10	0.56	0.07	0.69	−4.1	0.67	−4.0	1.20	0.95
Item 11	0.54	0.07	0.84	−2.0	0.84	−1.7	4.50	0.48
Item 12	0.53	0.07	0.79	−2.7	0.77	−2.7	6.10	0.30
Item 13	0.03	0.07	0.85	−1.8	0.86	−1.4	4.20	0.52
Item 14	0.12	0.07	0.73	−3.4	0.78	−2.2	4.20	0.52
Item 15	−0.80	0.09	1.08	0.8	0.86	−0.9	1.70	0.89
Item 16	−0.62	0.08	0.87	−1.4	0.79	−1.6	3.50	0.63
Item 17	−0.42	0.08	0.76	−2.8	0.68	−2.8	4.30	0.54
Item 18	−0.77	0.09	0.76	−2.6	0.68	−2.4	4.10	0.49
Item 19	0.14	0.07	0.92	0.12	1.57	0.20	2.23	0.28

*b* represents the item difficulty. MNSQ indicates the mean square fit statistics, and ZSTD is standardized as Z-scare fit statistics.

##### Evidence based on consequences

Based on DIF analysis, except for item 2 (i.e., even though bad things sometimes happen to me, I think life is beautiful), the difficulty of which was considered to differ by gender, there was no difference between boys and girls in any of the items. This can be interpreted as indicating that the probability of responding to the item was lower in boys than girls, although both groups actually possessed an identical level of MIL. In other words, even though boys and girls choose the same responses to Item 2, there is a high probability that their latent levels on MIL are different.

#### Reliability and item characteristics

**Figure 4:**
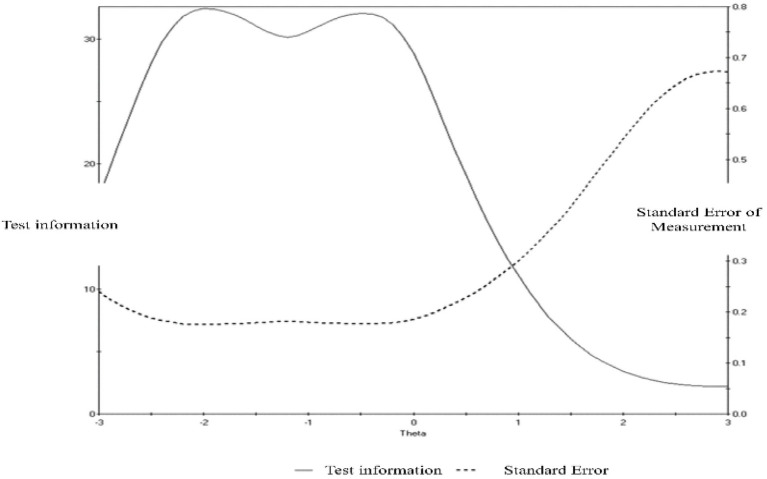
Test information and standard error of measurement of the K-MIL-CQ.

The Cronbach’s alpha of the final K-MIL-CQ was 0.93, and the coefficients of the individual subscales were 0.84 for attitude, 0.88 for creative, and 0.87, for experience. Figure 4 shows the test information curve (TIC) and SEM, indicating a certain range of levels where the MIL scale provides the highest information and lowest standard error. The graph indicates that the K-MIL-CQ provides the most reliable score for children aged 10–11, who have an average level of MIL.

## Discussion

The aim of this study was to validate K-MIL-CQ in South Korean elementary school students. We collected validity evidence based on content, factor structure, related measures, response patterns, and consequences. Additionally, we evaluated the scale’s item characteristics and reliability. Overall, the results suggest that while the K-MIL-CQ is a useful instrument with good psychometric properties, it requires minor modifications for optimal use in the South Korean context. The final version of the K-MIL-CQ includes a total of 18 items across three subscales: six items for attitude, seven items for creative, and five items for experience.

The K-MIL-CQ showed good content validity. According to the experts’ evaluation, each subscale appropriately represented the construct of MIL, and almost every item appropriately reflected the subscale it belonged to. However, three out of the five experts expressed some concerns about the clarity of items 7, 17, and 20, pointing out that the words “contribute” (item 7), “beauty in life” (item 17), and “God or higher power” (item 20) could be difficult for elementary school students to understand. Item 20 was removed based on the results of EFA analysis. To make the item easier for elementary school to understand, the word “contribute” in Item 7 was supplemented with the addition of the word “help” in parentheses. Based on the expert comment on item 17, we decided to incorporate concrete examples into the final version to make it easier to understand. As item 17 represents experiencing MIL through positive human interactions, engaging in significant events, and appreciating transcendent states or entities such as nature, art, or beauty (Shoshani and Russo-Netzer, 2017), examples such as “going to a beautiful park,” “looking at trees and flowers,” “watching good movies,” and “having a good time with family or friends” were included in the final version of the K-MIL-CQ.

In this study, we conducted EFA, based on which it was determined that like the original tool, the K-MIL-CQ has a stable three-factor structure. However, three items (one from the creative factor and two from the experience factor) were not included in the final K-MIL-CQ considering the statistical results as well as theoretical and cultural background. Regarding the creative subscale, Item 8, “I like taking the time to do important and meaningful things,” was removed because it loaded to all three factors. Among Korean children, “doing important and meaningful things” does not seem to strongly represent one of the three pathways to achieve MIL. The following items were removed from the experience subscale: “I feel connected to God or a higher power that gives me guidance” (Item 20) and “Being in nature makes me happy and calm” (Item 21). It is possible that South Korean elementary school students are unfamiliar with the concepts of guidance from God or a higher power and being in nature. Indeed, the Korean Meaning of Life Scale (Kang et al., 2007) does not include these concepts, suggesting that they are not commonly used to represent the experience of MIL in this population, at least in the South Korean context. Indeed, the Korean Meaning of Life Scale for school-aged children in Grades 4–6 (Kang et al., 2007) has extracted five factors, namely relational experience, positive attitude, satisfaction/hope, pursuit of goals, and experience of family love, but does not explicitly include themes or words related to self-transcendence, such as God, higher power, nature, universe, or spirituality. Similarly, the results of the content analysis of South Korean elementary school students’ experience of MIL (Kang, 2017) did not identify these themes, although the included themes reflected many kinds of ordinary daily activities such as “playing games,” “listening to music,” “practicing dancing,” “watching movies,” and “reading books.” Another content analysis of MIL conducted among 1,790 upper-grade elementary school students (Kim et al., 2010) identified various themes related to engaging in meaningful behavior, doing morally correct things, maintaining comfortable emotions, receiving help from others, serving others, having good relationships with friends, obeying parents’ guidance, being dutiful to one’s parents, staying with one’s parents, studying hard, becoming a great person, embodying diligence, and realizing achievements through studying; however, the responses related to self-transcendence were neither rich nor salient. The concepts of God, a supreme power, and nature represent important transcendent features through which people discover or cultivate a sense of MIL (Frankl, 1959; Steger, 2012). South Korean adolescents might be able to enrich their MIL experience by becoming more familiar with these sources of MIL.

It is also notable that themes related to obeying one’s parents, meeting familial expectations, and achieving academic and career success were commonly extracted from Korean children’s answers to the question of what makes their lives meaningful. Similarly, in a study on the development of a multidimensional meaning of life scale using a sample of Korean university students, academic and career success and recognition from others were identified as sources of meaning (Park and Kwon, 2012). It seems that experiencing life’s meaning through achieving one’s goals (especially those related to academic excellence) is familiar, while enjoying nature or pursuing transcendence is less appreciated as a meaning-making pathway. Although a strong emphasis on the category of doing things (e.g., achievement, academic excellence, test results) to experience MIL might reflect the specific Korean cultural context, Korean children may also benefit from expanded opportunities to cultivate MIL through experiential and attitudinal pathways because these three distinctive pathways could compensate for each aspect, ultimately enriching individuals’ MIL experience.

Corresponding to the original scale, a hierarchical second-order model with three factors (attitude, creativity, and experience) as first-order factors and MIL as a second-order factor was confirmed. This result implies that Korean children seem to perceive MIL as a multidimensional psychological construct that can be achieved in a variety of ways, similar to the original scale. This provides a good rationale for emphasizing three distinctive conceptual pathways for cultivating MIL among Korean children. As expected from theories and previous empirical studies, students with higher levels of MIL showed higher levels of subjective well-being, as measured by life satisfaction, positive affectivity, and higher levels of self-esteem. These results are consistent with previous studies showing positive associations between MIL and indicators of positive well-being and self-evaluation (e.g., Steger et al., 2006; Kang et al., 2007; Owens et al., 2009; Shoshani and Russo-Netzer, 2017). These results support that K-MIL-CQ is a valid instrument for measuring children’s MIL. Based on the results of the response pattern analyses of the infit and outfit statistics, all items were appropriate for measuring the construct of MIL. In addition, the most of PRFs in each item showed that the intervals between adjacent categories provided relatively meaningful information. The results of DIF analysis based on gender indicated that boys had a lower probability of responding to item 2 (even though bad things sometimes happen to me, I think life is beautiful) than girls at the same level of MIL. In other words, although boys and girls had the same level of MIL, their responses to Item 2 could be different. Thus, item 2 might not be a good indicator to investigate gender differences in MIL because it does not effectively differentiate levels of MIL in terms of gender. Attention is needed to interpret the score of item 2 from a gender-related perspective. We suspect that the word “beautiful” is more familiar to girls, but more research is needed to examine what contributes to the differential probability of responding to the item. Finally, the K-MIL-CQ showed good internal consistency, indicating that the instrument could serve as a reliable tool for assessing MIL in South Korean children. The current study provides evidence of the validity and reliability of the K-MIL-CQ in elementary school students. Based on the psychometric properties in this study, we suggest that the K-MIL-CQ is a useful instrument for measuring the three pathways of Frankl’s triangle, not overlapping with other similar constructs. The current study will help researchers, educators, and practitioners better understand the MIL experience of early adolescents by facilitating empirical research on this topic.

## Limitations and future study

This study has several limitations that should be taken into consideration. First, it was based on a relatively small sample of 10–11-year-old students. Future research should include elementary school students in lower grades to evaluate whether the instrument is applicable to them. As the original MIL-CQ was developed for children aged 9–12 years, we expect that K-MIL-CQ may also be applicable to younger children; however, some modifications may be needed. Second, to assess correlations with other measures, the current study only included tools assessing subjective well-being and self-esteem. Future studies need to examine whether the instrument shows validity evidence in terms of relations with negative indices of mental health and well-being by including scales measuring psychological and behavioral difficulties and problems. Finally, this study only focused on a South Korean sample with 277 elementary school students. Although the sample size was sufficient to obtain valid results from the statistical analyses conducted in this study, a larger sample would improve the generalizability of the results. In interpreting the results of the current study and comparing them with those obtained from the original MIL-CQ, which was developed in another country, the authors have made several speculations regarding students’ understanding of phrasing, cultural differences, and gender differences. Future studies must utilize in-depth interviews with young South Korean adolescents to examine the validity of these hypotheses. Further, interviews with parents and teachers will be helpful in obtaining a more comprehensive picture of how family, school, and society facilitate potential pathways of experiencing MIL among young adolescents.

## Data availability statement

The raw data supporting the conclusions of this article will be made available by the authors, without undue reservation.

## Ethics statement

Ethical review and approval was not required for the study on human participants in accordance with the local legislation and institutional requirements. Written informed consent to participate in this study was provided by the participants' legal guardian/next of kin.

## Author contributions

YC and JS: conceptualization, methodology, data analysis, and writing. All authors contributed to the article and approved the submitted version.

## Funding

This work was supported by the Ministry of Education of the Republic of Korea and the National Research Foundation of Korea (NRF-2019S1A5A2A03052192).

## Conflict of interest

The authors declare that the research was conducted in the absence of any commercial or financial relationships that could be construed as a potential conflict of interest.

## Publisher’s note

All claims expressed in this article are solely those of the authors and do not necessarily represent those of their affiliated organizations, or those of the publisher, the editors and the reviewers. Any product that may be evaluated in this article, or claim that may be made by its manufacturer, is not guaranteed or endorsed by the publisher.
